# Community based study to compare the incidence and health services utilization pyramid for gastrointestinal, respiratory and dermal symptoms

**DOI:** 10.1186/1472-6963-12-211

**Published:** 2012-07-23

**Authors:** Nusrat Najnin, Martha Sinclair, Andrew Forbes, Karin Leder

**Affiliations:** 1 Department of Epidemiology and Preventive Medicine, School of Public Health and Preventive Medicine, Monash University, Melbourne, Australia

**Keywords:** Respiratory symptoms, Gastrointestinal symptoms, Dermal symptoms, Burden of illness, Healthcare utilization

## Abstract

**Background:**

Gastrointestinal (GI), respiratory and dermal symptoms are common and cause substantial morbidity, although the information on their exact incidence and comparative burden is limited. The aim of this study was to describe the epidemiology and rate these three major symptom complexes in order to improve our understanding of the health burden imposed by these symptoms.

**Methods:**

We used data from a community based randomised control trial conducted from June 2007 to August 2008 among 277 South Australian families consuming rainwater. Using weekly health diaries, we prospectively collected information on GI (diarrhoea or vomiting), respiratory (sore throat, runny nose or cough) and dermal (rash, generalised itch or dermal infection) symptoms, as well as on relevant GP visits, time off work and/or hospitalisation due to these symptoms. Data were analysed using generalized estimating equations approach taking into account the variable number of weeks of follow-up of each individual and within-family clustering of responses.

**Results:**

Over one year, at least one episode of GI symptoms was reported by 54% of participants (95% CI 50%-58%), at least one respiratory episode by 91% (95% CI 88%-93%) and at least one episode of dermal symptoms by 27% (95% CI 24%-30%). The average number of weeks per year during which respiratory symptoms occurred was four times greater than for GI or dermal symptoms (4.9, 1.2 and 1.2 weeks, respectively, p<0.001), with an average number of GP visits per person per year being twice as frequent (0.48, 0.26, 0.19 respectively, p<0.001). However, on a per episode basis, a higher proportion of people saw a GP or were hospitalised for GI symptoms.

**Conclusions:**

This first comparative study of three different symptom complexes showed that although respiratory symptoms are most common, GI symptoms cause a greater per episode burden on healthcare resources. Measuring and comparing the community based burden of these symptom complexes will assist evidence-based allocation of resources.

## Background

Infectious gastrointestinal (GI), respiratory and dermal diseases cause substantial morbidity and economic loss [1-4]. However, there is limited information available on the incidence and comparative burden of illness related to these three symptom complexes. There is also minimal information on the proportions of people suffering from these symptoms who take time off work, visit a doctor, or are hospitalized. Collection of data regarding incidence of symptoms at the community level is difficult and resource-intensive, and is generally done during dedicated studies (e.g. via health diaries). Better understanding of the incidence, morbidity, and healthcare seeking behaviour associated with GI, respiratory and skin diseases would be useful for appropriate allocation of health resources.

The aim of this study is to describe the epidemiology and rate of disease symptoms related to gastrointestinal, respiratory and dermal systems among 277 Australian families and to compare the health care seeking pattern for these three major symptom complexes in order to improve our understanding of the health burden imposed by these symptoms.

## Methods

### Study participants and data collection

As part of a double-blinded, randomized, controlled trial conducted in South Australia from June 2007 to August 2008, weekly health diaries were administered to 300 families to collect data over a 12 month period. Eligibility criteria for inclusion related to the main study hypothesis, which was to determine whether consumption of untreated rainwater contributed to gastroenteritis. The criteria included: using untreated rainwater from an above-ground tank as the normal drinking water source; having at least four eligible household members (including at least 2 children aged 1 to 15 years); home ownership or stable rental history (12 months or more in current home); and having a reasonable command of English. Households were randomly allocated real or sham water treatment devices to treat rainwater for drinking; real devices removed microorganisms from the water, sham devices did not [5].

The health diary included reporting of symptoms related to gastrointestinal (GI), respiratory and dermal complaints on weekly basis. For each of the reported symptoms, the participants also noted if they consulted a doctor, if they or any of their family members had to take time off for the symptom event, or if they were hospitalized.

We defined a GI symptom as people reporting either passing a loose stool or vomiting at least once within 24 hours. We considered people to have respiratory symptoms if they had either sore throat or runny nose or cough. If people reported either rash, generalised itching or dermal infection, then we defined them as having dermal symptoms.

Rather than collecting information of each of these symptom complexes every day, we collected an overall presence or absence of each type of clinical event over the course of each week. Similarly, if any study participant visited a doctor or was hospitalized in a particular week, we asked them to mark that week in their health diary. However, we collected information on a daily basis for time off from school or work, or inability to perform the normal duties because of illness or to take care of someone who was unwell.

### Data management

Completed health diaries were mailed to the Study Centre (Monash University) every 4 weeks. Diaries were scanned, and the accuracy and completeness of data was verified using the Cardiff Teleform software (version 10.1, 2006; Vista, California, USA) before data entry into a Microsoft Access® database. Reporting participants were telephoned for clarification if information was missing or ambiguous.

### Data analysis

The number of weeks (or days where applicable) with valid information was determined for each of the three symptom complexes, associated doctor and hospital visits, and time off work. Similarly, for analysing the seasonality of the reported symptoms, we considered only the number of weeks with valid information on the symptoms. We categorised the data collection period into four different seasons. Seasons were defined as follows: spring, September to November; summer, December to February; autumn, March to May; and winter, June to August.

We estimated the proportion of subjects experiencing at least one episode of each symptom complex over the one year period, taking into account the variable number of weeks of follow-up of each individual and within-family clustering of responses. We used a generalized estimating (GEE) approach with a complementary log-log link function, offset term as the logarithm of the number of weeks with valid information, exchangeable correlation structure and robust standard errors [6]. This approach is based on the assumption that the probability of an episode in a given week is a constant value and that episodes occurring from one week to the next are independent. The same approaches were used for estimating proportions with doctor visits or hospitalizations over the one year period. Proportions experiencing episodes across seasons were estimated similarly using the number of weeks of information per season in the offset term.

The average number of weeks with GI, respiratory and dermal symptoms, doctor visits and hospitalizations per person per year were estimated by an analogous GEE approach using the Poisson distribution with logarithmic link function and the same offset term to accommodate differing number of weeks of information. The comparison between the three symptom complexes was performed using GEE in which the three complexes were input as repeated measurements within each person with associated indicator predictor variables.

Results are presented as estimated proportions or average number of weeks, or rate ratios for symptom complexes or health care utilization with 95% confidence intervals. All calculations were performed using Stata version 11.1 [7].

### Ethical considerations

During enrolment, written informed consent was obtained from all adult household members and from parents and guardians on behalf of children. All results presented in this study received approval from the Monash University Standing Committee on Ethics in Research Involving Humans (SCERH; 2006/555EA) and the South Australia Department of Health Human Research Ethics Committee.

## Results

The original study had 300 households with 1,352 residents. From these we excluded 23 households with missing demographic information or who provided no information on the three symptom complexes of interest. Therefore our analysis included 277 households with 1,237 participants. Comparison of gastroenteritis rates between groups with real or sham water treatment devices showed no significant difference, indicating that drinking untreated rainwater did not contribute appreciably to health outcomes [5]. This cohort can thus be considered generally representative of households with children.

Eleven percent (n = 132) of the study participants were children ≤5 years of age. The numbers of male and female participants in the study were similar (Table 1).

**Table 1:** Demographic characteristics of study participants

**Characteristics of people**	**N = 1237 (%)**
**Age**	
≤5 years	132 (11)
>5 to ≤15 years	489 (40)
>15 years	616 (50)
**Sex**	
Males	626 (51)
Female	611 (49)
Educational status*	
Attending childcare/preschool	98 (8)
Primary	413 (33)
Secondary/Commercial/Technical	320 (26)
College/University	368 (30)

* Educational status: 38 (3%) missing data.

**Figure 1:**
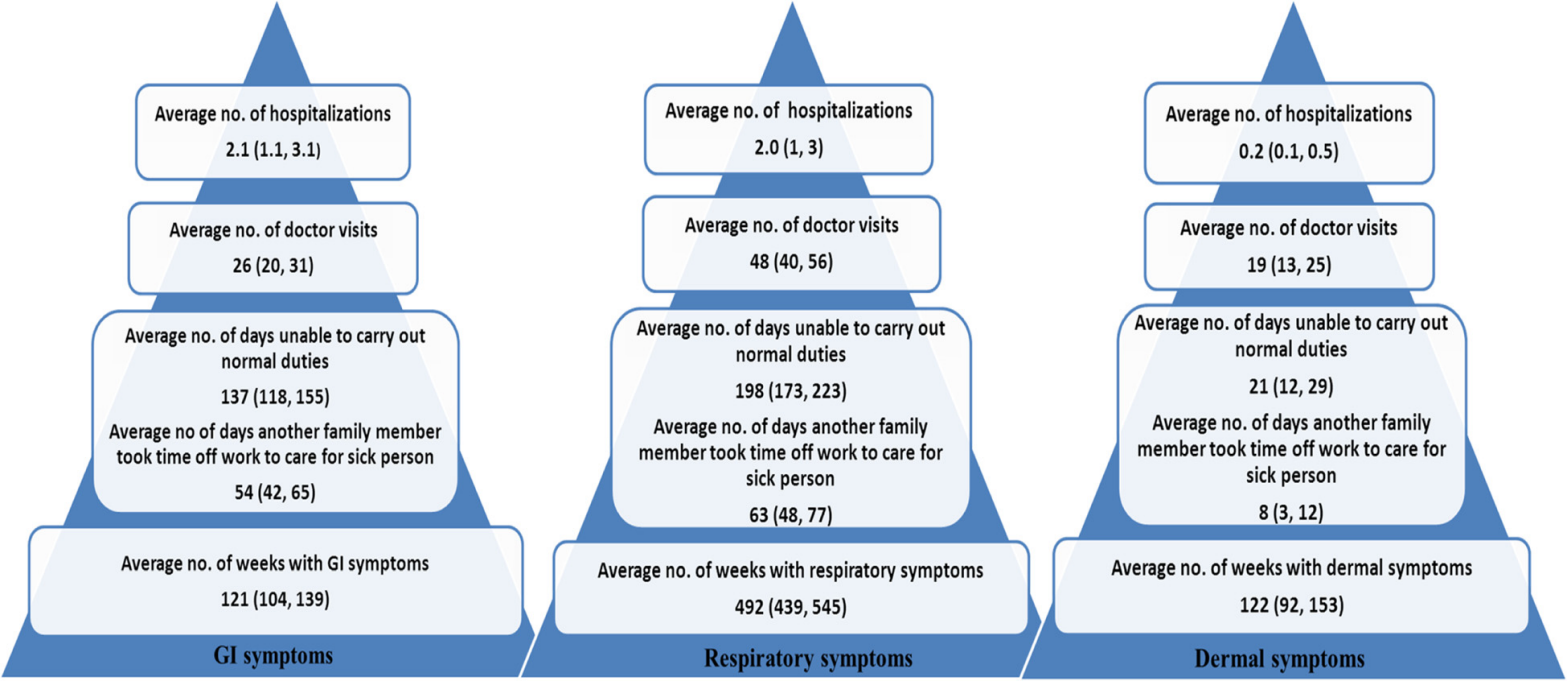
**Burden of GI, respiratory and dermal symptoms in the community among the study participants.** The results are presented as frequencies per 100 person-years, with the 95% confidence intervals shown in brackets.

The estimated proportion of study participants experiencing GI symptoms at least once over the one year period was 54% (95% CI: 50, 58), with 15% (95% CI: 13, 18) reporting visiting a doctor for these symptoms, and 2% (95% CI: 1, 3) hospitalized (Table 2). The number of weeks with reported GI symptoms ranged from 0–27. On average, an estimated 1.21 weeks (95% CI: 1.04, 1.39) with GI symptoms were reported per person per year (Figure 1).

**Table 2:** Estimated proportion of people experiencing at least one event of GI, respiratory or dermal symptoms in a year and relevant estimated care seeking pattern (n = 1237)*

**Disease symptoms and characteristics of participants**	**GI symptoms % (95% CI)**	**Respiratory symptoms % (95% CI)**	**Dermal symptoms % (95% CI)**
**Proportion of study participants with symptoms**			
Age category (years)			
≤5 years	81 (74, 87)	97 (94, 99)	53 (43, 63)
>5 to ≤15 years	54 (49, 60)	93 (90, 96)	31 (26, 36)
>15 years	48 (43, 53)	87 (83, 90)	18 (15, 22)
Total	54 (50, 58)	91 (88, 93)	27 (24, 30)
**Proportion of study participants who visited a doctor**			
≤5 years	38 (28, 48)	62 (51, 72)	24 (15, 32)
>5 to ≤15 years	14 (11, 18)	24 (20, 29)	12 (8, 15)
>15 years	11 (8, 14)	24 (20, 28)	6 (3, 8)
Total	15 (13, 18)	29 (25, 32)	10 (9, 12)
**Proportion of study participants who were hospitalized**	2 (1, 3)	2 (1, 3)	0.2 (0.01, 0.1)
**Proportion of study participants who took time off school/work/child care or who were unable to carry out normal duties**	32 (29, 36)	52 (48, 56)	6 (4, 7)
**Proportion of events for which a family member took time off work/school to care for the sick person**	16 (13, 19)	21 (18, 24)	3 (2, 4)

*Because of dropouts the number of observations varied from 1237 to 1228.

An estimated 91% (95% CI: 88, 93) of participants experienced at least one episode of respiratory symptoms during the study period, with 29% (95% CI: 25, 32) visiting a doctor and 2% (95% CI: 1, 3) being hospitalized at least once (Table 2). The number of weeks with reported respiratory symptoms ranged from 0–36. On average, an estimated 4.92 weeks (95% CI: 4.39, 5.45) with respiratory symptoms were reported per person per year (Figure 1).

An estimated 27% (95% CI: 24, 30) experienced dermal symptoms at least once during the study period. These symptoms caused an estimated 10% (95% CI: 9, 12) of participants to visit a doctor and 0.2% (95% CI: 0.01, 0.1) to be hospitalized at least once (Table 2). The number of weeks reported with dermal symptoms ranged from 0–51 among study participants, and participants suffered from an estimated average of 1.22 (95% CI: 0.92, 1.53) weeks with dermal symptoms per year (Figure 1).

There was variation in symptom reporting across age for each complex (all p<0.001). Children ≤5 years of age suffered most frequently from each of the three symptom complexes, and were also most likely to visit a doctor because of these symptoms (Table 2). The estimated average number of weeks per year during which symptoms occurred varied across the three symptom complexes (p<0.001). The number of weeks for which respiratory symptoms occurred was four times more than for GI symptoms [rate ratio (RR) 3.9; 95% CI: 3.5, 4.5] or dermal symptoms (RR 3.9, 95% CI: 3.1, 4.9). The estimated average number of weeks people suffered from dermal symptoms per year was similar to GI symptoms (rate ratio 1.0; 95% CI: 0.8, 1.3) (Table 3).

**Table 3:** **Comparisons of rates (rate ratio) of symptom complexes and associated health care-seeking pattern with 95% confidence intervals (CIs) and*p*values among the study participants**

**Reported symptoms and associated care-seeking pattern**	**Rate ratio**	**95% CI**	***p*value**
**Reported symptoms**			
GI (reference category)	1.0	-	<0.001
Respiratory	3.9	3.5, 4.5	
Dermal	1.0	0.8, 1.3	
**Doctor visits**			
GI (reference category)	1.0	-	<0.001
Respiratory	1.9	1.5, 2.3	
Dermal	0.7	0.5, 1.1	
**Hospitalization**			
GI (reference category)	1.0	-	0.006
Respiratory	0.9	0.5, 1.6	
Dermal	0.1	0.03, 0.4	

The pattern of presentation to primary care doctors across the three symptom complexes varied similarly (p<0.001). However, on exploratory analysis we found that the clinical episodes leading to doctor visits for respiratory symptoms occurred at only twice the rate of GI or dermal symptoms (RR 1.9, 95% CI: 1.5, 2.3 compared to GI; and RR 2.5, 95% CI: 1.8, 3.4 compared to dermal). This indicates that, even though people were four times more likely to get respiratory symptoms compared to the other two symptom complexes, proportionately more people actually visited a doctor per episode if they had GI or dermal symptoms. The estimated average number of hospitalizations per person per year also varied across the symptom complexes (p = 0.006) (Table 3). The burden of disease in terms of hospitalization was similar for both respiratory and GI symptoms, but was lower for dermal symptoms (RR 0.1 with significant 95% CIs for dermal compared to both GI and respiratory symptoms). On further exploratory analysis we found that one estimated doctor visit occurred for every five weeks of GI symptoms (95% CI: 4.0, 6.5), ten weeks of respiratory symptoms (95% CI: 8.3, 11.3) and seven weeks of dermal symptoms (95% CI: 4.8, 9.0). One estimated hospitalization was reported for every 56 weeks for GI symptoms (95% CI: 34.2, 91.0), 243 weeks of respiratory symptoms (95% CI: 151.0, 391.3) and 582 weeks of dermal symptoms (95% CI: 143.2, 2365.0). Compared to respiratory symptoms, GI complaints lead to more frequent doctor visits and more frequent hospitalizations per episode. Compared to respiratory symptoms, dermal complaints lead to more frequent doctor visits but less frequent hospitalizations per episode.

The estimated average number of days per year taken off work because of respiratory symptoms was 1.5 times more (95% CI: 1.3, 1.6) than for GI symptoms, and for dermal symptoms the estimated average number of days taken off work per year was lower than for GI symptoms (rate ratio 0.2; 95% CI: 0.1, 0.2). The same comparative results were found for time taken off work to look after unwell family members.

**Figure 2:**
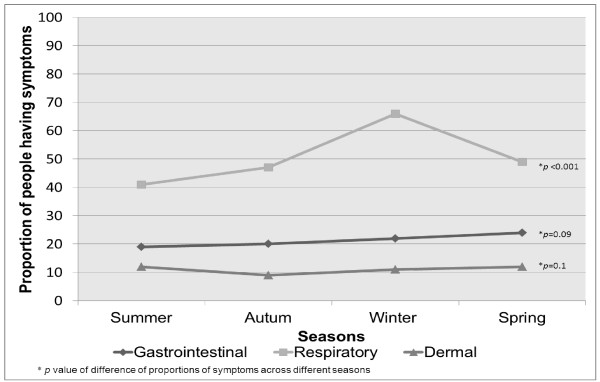
Proportion of people experiencing at least one episode of symptoms across different seasons during the study period.

There was seasonal variation in the frequency of respiratory symptoms (p <0.001) with a marked increase in frequency in winter, but we found no seasonal variations for GI or dermal symptoms (p = 0.09 for GI and p = 0.10 for dermal) (Figure 2).

## Discussion

To our knowledge, this study represents the first time the community-based burden of three different symptom complexes has been assessed and compared through a prospective cohort study. Our findings suggest that community GI, respiratory and dermal symptoms are common and represent a significant burden of illness. Because our data were all from the same cohort, it gave us a unique opportunity to compare the absolute number of weeks with symptoms, and the absolute and relative frequency of doctor visits, time-off work and hospitalization for each symptom complex.

The proportions of people reporting respiratory and dermal symptoms at least once per year are consistent with other studies [2,4,8]. Comparison of the three symptom complexes showed that respiratory symptoms imposed the highest burden in terms of symptom frequency, absolute numbers of doctor visits, and absolute number of days taken off work. Similar proportions of participants with GI or dermal symptoms visited a doctor, but dermal episodes were associated with the lowest proportion of people reporting time off work or hospitalization. While respiratory symptoms were reported four times more commonly than GI symptoms, time off work and doctor visits were only twice as frequent compared to respiratory symptoms, and equal numbers of people reported hospitalization for GI and respiratory symptoms per year. Thus, although respiratory symptoms were most common overall, on a per episode basis, participants with GI symptoms were more likely to see a doctor, take time off work, or be hospitalized. This infers that the overall burden in terms of lost productivity and use of health care services is greatest from respiratory symptoms, but the burden per episode is greatest for diarrhoea and/or vomiting, thereby suggesting that the severity and impact of GI symptoms is greater.

Because the focus of the original study was GI symptoms, it is possible that some differential reporting of GI versus the other symptoms may have occurred, and that GI symptoms may have been more likely to prompt a visit to a doctor among study participants than either respiratory or dermal symptoms. However the rate of gastroenteritis that we found in this population (0.77 episodes/person/year) is similar to that seen in other Australian studies [1,5,9,10], indicating that study participants were not more sensitive to and did not differentially report GI symptoms compared to the other two symptom complexes. This also suggests that the fact that study participants were rainwater drinkers is unlikely to impact on the generalizability of our study findings [5].

People suffered most from respiratory symptoms during winter. This is consistent with findings from other studies performed in similar contexts [11]. The possible reasons for greater numbers of respiratory symptoms during winter are increased indoor and outdoor air pollution during this season [12-15] as well as (presumed) higher rates of respiratory infections.

Our study has a number of limitations. Firstly, we relied on self-reported data over a one year time period. It is possible that response fatigue may have meant some people did not report all symptoms, which may have resulted in under-reporting and therefore underestimation of symptom incidence. However, it is unlikely that this would have had a major effect on the comparative frequency of the different symptom profiles. We analysed results according to weeks with symptoms, but acknowledge that respiratory symptoms last longer on average than GI symptoms [2]. It is therefore possible that GI symptoms over two weeks belonged to two different episodes whereas respiratory symptoms over two weeks may have belonged to the same episode. This may have led to a slight over-estimation of the difference in the health care access for GI compared to respiratory symptoms.

Secondly, we collected information about the presence or absence of different symptoms using very broad symptom-based case definitions. Therefore the results have to be interpreted with caution, as, not all symptoms were necessarily serious or infectious. Thirdly, our findings may not be generalizable across the whole community. We deliberately enrolled selected English-speaking households in South Australia with at least two children aged 1–15 years who were drinking untreated rainwater. Therefore, our results reflect the demographics of those included, namely young families living in urban Adelaide, and may not be applicable to other populations. It is possible that the inclusion of young children in the study may have resulted in a higher number of illness episodes and doctor visits per person per year. Additionally, the results are indicative of health care seeking behaviour in an Australian population and may not apply to countries with different health care structures. Nevertheless, we believe our findings on the relative use of services for these different conditions are likely to apply to other developed nations.

Finally, even though we calculated the ‘average number of weeks’ people visited doctors or were hospitalized per person per year, we interpreted the data as ‘average number’ of doctor visits and hospitalizations per person per year. It is possible that people might have visited a doctor more than once in a week, in which case we might have underestimated the number of doctor visits. However, this would not be common and therefore should not affect our interpretations. Similarly, it is highly unlikely that anyone would be hospitalized more than once in a week.

## Conclusions

In summary, in a prospective cohort of 277 Australian families, GI, respiratory and dermal symptoms were a major cause of morbidity and contributed significantly to doctor visits and to time off work. Respiratory symptoms occurred most commonly, but GI symptoms led to the greatest impact on health care visits. This comparative symptom-based data analysis focusing on a number of markers of illness severity provides novel information regarding health care resource utilization and absenteeism that is attributable to GI, respiratory and dermal symptoms. This adds to our understanding of the direct health care costs as well as the indirect costs of these symptoms to society.

## Abbreviations

GI, Gastrointestinal; GEE, Generalised estimating equations; RR, Rate ratio; CI, Confidence interval.

## Competing interests

The authors declare that they have no competing interest.

## Authors’ contributions

NN analysed and interpreted the data and prepared the draft manuscript; MS contributed in conception and design of the study, revised the manuscript critically for important intellectual content; AF contributed in analysing and interpreting the data, revised the manuscript critically for important intellectual content; KL contributed in conception and design of the study, interpreted the data, revised the manuscript critically for important intellectual content, gave final approval of the version submitted to the journal. All authors read and approved the final manuscript.

## Pre-publication history

The pre-publication history for this paper can be accessed here:

http://www.biomedcentral.com/1472-6963/12/211/prepub
